# Early Stimulation of TREK Channel Transcription and Activity Induced by Oxaliplatin-Dependent Cytosolic Acidification

**DOI:** 10.3390/ijms21197164

**Published:** 2020-09-28

**Authors:** Marianna Dionisi, Federico Alessandro Ruffinatti, Beatrice Riva, Dmitry Lim, Annalisa Canta, Cristina Meregalli, Giulia Fumagalli, Laura Monza, Antonio Ferrer-Montiel, Asia Fernandez-Carvajal, Guido Cavaletti, Armando A. Genazzani, Carla Distasi

**Affiliations:** 1 Department of Pharmaceutical Sciences, University of Piemonte Orientale, Via Bovio 6, 28100 Novara, Italy; marianna.dionisi@uniupo.it (M.D.); federicoalessandro.ruffinatti@uniupo.it (F.A.R.); beatrice.riva@uniupo.it (B.R.); dmitry.lim@uniupo.it (D.L.); armando.genazzani@uniupo.it (A.A.G.); 2 Experimental Neurology Unit, School of Medicine and Surgery, University of Milano-Bicocca, Via Cadore 48, 20900 Monza, Italy; annalisa.canta@unimib.it (A.C.); cristina.meregalli@unimib.it (C.M.); giulia.fumagalli1@unimib.it (G.F.); laura.monza@unimib.it (L.M.); guido.cavaletti@unimib.it (G.C.); 3 Biología Molecular y Celular, Universidad Miguel Hernández de Elche, 03202 Elche (Alicante), Spain; aferrer@umh.es (A.F.-M.); asia.fernandez@umh.es (A.F.-C.)

**Keywords:** oxaliplatin, TREK channels, neuropathic pain, pH, DRG neurons, Na^+^/H^+^ exchanger, electrophysiology, TRPV1

## Abstract

Oxaliplatin-induced peripheral neuropathy is characterized by an acute hyperexcitability syndrome triggered/exacerbated by cold. The mechanisms underlying oxaliplatin-induced peripheral neuropathy are unclear, but the alteration of ion channel expression and activity plays a well-recognized central role. Recently, we found that oxaliplatin leads to cytosolic acidification in dorsal root ganglion (DRG) neurons. Here, we investigated the early impact of oxaliplatin on the proton-sensitive TREK potassium channels. Following a 6-h oxaliplatin treatment, both channels underwent a transcription upregulation that returned to control levels after 42 h. The overexpression of TREK channels was also observed after in vivo treatment in DRG cells from mice exposed to acute treatment with oxaliplatin. Moreover, both intracellular pH and TREK channel transcription were similarly regulated after incubation with amiloride, an inhibitor of the Na^+^/H^+^ exchanger. In addition, we studied the role of oxaliplatin-induced acidification on channel behavior, and, as expected, we observed a robust positive modulation of TREK channel activity. Finally, we focused on the impact of this complex modulation on capsaicin-evoked neuronal activity finding a transient decrease in the average firing rate following 6 h of oxaliplatin treatment. In conclusion, the early activation of TREK genes may represent a mechanism of protection against the oxaliplatin-related perturbation of neuronal excitability.

## 1. Introduction

Chemotherapy-induced peripheral neurotoxicity remains a common side effect of several anticancer agents, including vinca alkaloids, taxanes, platinum derivatives, bortezomib, and thalidomide [1]. Among these agents, oxaliplatin (OHP) induces an acute sensory neuropathy characterized by alterations of sensitivity, dysesthesias, paresthesias, and cramps, which are predominantly located in the extremities and the face and, in most patients, last for hours or even days after the first OHP injection. In addition, many OHP-treated patients who experience this acute syndrome also develop chronic sensorial symptoms that are also common to other platinum-based chemotherapies [2,3]. Currently, no effective agent for the prevention or the treatment of OHP-induced peripheral neurotoxicity (OIPN) exists, thus significantly reducing the quality of life of patients [1,4].

The pathogenesis of OIPN remains poorly understood. In this sense, OHP is known to induce hyperexcitability by remodeling the expression and/or altering the function of some molecular targets that participate in the transduction of the thermal and mechanical sensation at the peripheral level [2]. Among these, transient receptor potential (TRP) channels are generally accepted as primary receptors for the detection of both physiological and noxious temperatures [5,6]. Cumulative genetic and pharmacological evidences suggest the involvement of cold-activated TRP members in the onset of OIPN [2,7]. However, other channels are also known to modulate thermosensation. For instance, hyperpolarizing K^+^ two-pore domain (K2P) channels work as regulators of the excitability of primary afferent fibers, and thus of pain signaling by tuning the excitation elicited by several stimuli, including temperature [8,9,10] and mechanical forces [11]. In accordance with this idea, it has been proposed that all TREK-family channels (TREK-1, TREK-2, and TRAAK), which are widely expressed in dorsal root and trigeminal ganglia, are implicated in the mediation of cold hyperalgesia, cool allodynia, and mechanical hypersensitivity following the treatment with OHP [2,12].

Interestingly, recent reports have shown that therapeutically-relevant OHP concentrations induce cytosolic acidification in cultured dorsal root ganglion (DRG) neurons as well as in DRG cells from treated animals [13]. In in vitro experiments, acidification occurs after 30 min from OHP application [14] and produces a reversible hypersensitization of TRPA1 channels [13]. In the present study, we have investigated the early involvement of K2P channels, TREK-1 and TREK-2, in this context since these channels, among other stimuli, also respond to intracellular pH changes (pH_i_) [15,16,17]. We now report that therapeutically relevant OHP concentration treatment transiently increases the expression of TREK channels, both in vitro and in vivo. Interestingly, these channels undergo an upregulation following incubation with amiloride, a nonselective inhibitor of the Na^+^/H^+^ exchanger. Last, we show that the OHP-induced cytosolic acidification positively regulates the activity of TREK-2 channels. Overall, this complex modulation transiently reduces capsaicin-induced excitability, suggesting a possible short-term protective function of K2P.

## 2. Results

### 2.1. Effects of OHP on TREK Transcription in DRG Cells

**Figure 1:**
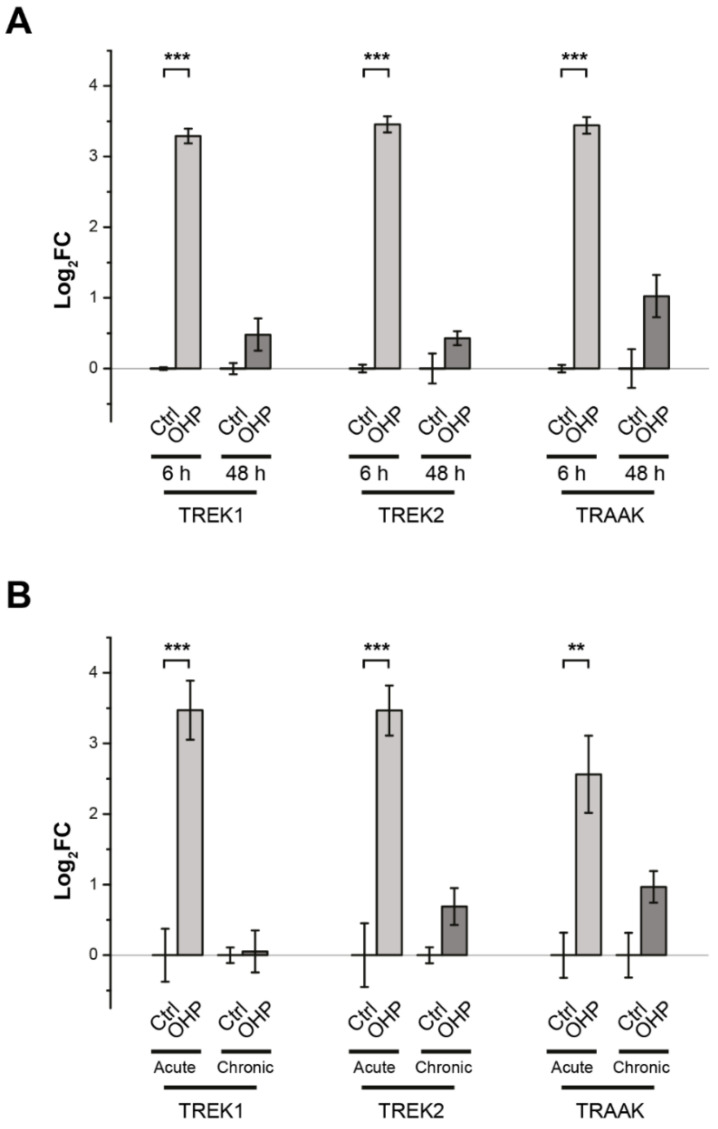
Oxaliplatin transiently affects the transcription of TREK-1, TREK-2, and TRAAK channels. Each bar represents the mean ± SEM of treated vs. control log_2_FC of gene expression, as measured through real-time quantitative PCR (RT-qPCR). (**A**) For each channel, oxaliplatin (OHP)-induced changes in mRNA level were measured in cultured dorsal root ganglion (DRG) cells after 6 h of treatment and 42 h later (*n* = 2 biological replicates, each in technical triplicate). (**B**) mRNA expression from DRG cells was measured upon in vivo acute and chronic OHP treatment and compared with the untreated counterpart (*n* = 4 biological replicates, each in technical triplicate). In both panels, controls are normalized to zero. ** *p*-value < 0.01, *** *p*-value < 0.001; Bonferroni-corrected multiple *t*-tests.

Previous data [2,18] has shown a drastic decrease in the expression of TREK channels in mice four days after treatment with a single dose of OHP, just at the peak of pain hypersensitivity [2]. To investigate the early effect of the treatment with OHP on the expression of TREK channels in DRG cells, we performed real-time quantitative PCR (RT-qPCR) both in vitro and in vivo. In in vitro experiments, the mRNA levels were measured after a 6 h treatment with OHP, i.e., immediately after removing the antineoplastic drug (OHP 6 h), and 42 h later (OHP 48 h). As shown in Figure 1A, TREK channels underwent a transient upregulation following OHP treatment. Furthermore, we also studied the effect of OHP on TRPV1 expression after 6 h of treatment and did not find any significant change (log_2_FC = 0.57 ± 0.23 relative to the untreated control, *p*-value = 0.19, Student’s *t*-test), as already reported for OHP-treated neurons after 48 h from plating [13]. These data indicate a relative specificity of action for OHP in gene expression modulation.

In in vivo experiments, mice were treated either with OHP twice a week for four weeks (chronic condition) or only once for 24 h (acute condition). TREK mRNA levels were measured 24 h after the last treatment. We observed that the acute treatment with OHP induced a significant increase in the mRNA levels of TREK-1 and TREK-2 channels (Figure 1B).

### 2.2. Effects of Amiloride on Intracellular pH and TREK Channel Transcription

**Figure 2:**
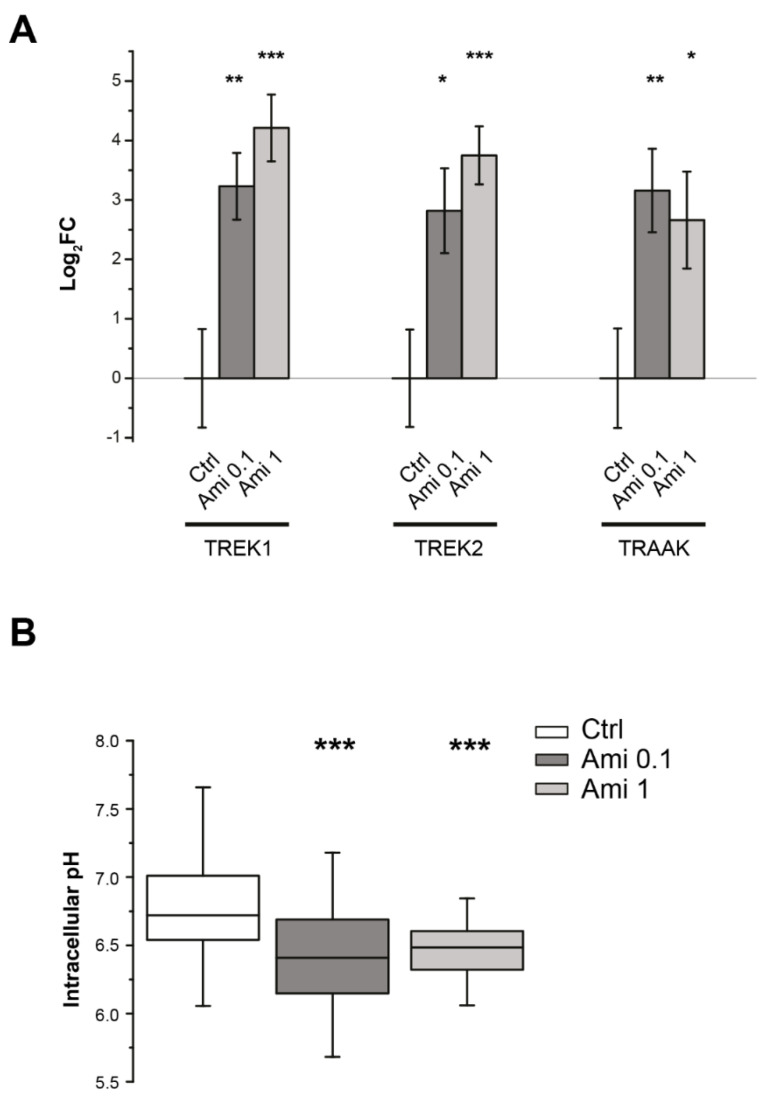
Effects of amiloride on DRG cells. (**A**) Each bar represents the mean ± SEM of treated vs. control log_2_FC of gene expression, as measured through RT-qPCR. Data show a significant increase in all the K^+^ two-pore domain (K2P) channels considered in this analysis after 6 h of treatment with two different concentrations of amiloride (0.1 and 1 µM). The transcriptional effects of amiloride were assessed by testing the log_2_FC of each experimental condition against its reference control value normalized to zero. In each condition, experiments were performed in *n* = 4 biological replicates (each in technical triplicate). * *p*-value < 0.05, ** *p*-value < 0.01, *** *p*-value < 0.001; ANOVA and Dunnett’s post hoc test for each channel separately. (**B**) Blockade of Na^+^/H^+^ exchanger (NHE) with 0.1–1 µM amiloride significantly decreased intracellular pH in DRG neurons (each box represents 100 to 150 cells from *n* = 4 independent experiments; *** *p*-value < 0.001; ANOVA and Dunnett’s post hoc test).

Riva et al. (2018) reported that 6 h of treatment with OHP was sufficient to reduce cytosolic pH levels in DRG neurons significantly. To investigate whether the changes in intracellular pH induced by OHP treatment can be responsible for the upregulation of TREK mRNA in DRG cells, we performed RT-qPCR after a 6 h treatment with amiloride, a stimulus that is known to promote intracellular acidification [19]. This is, indeed, a nonselective inhibitor of the Na^+^/H^+^ exchanger (NHE), an important class of transporters involved in the intracellular pH homeostasis [20,21]. The mRNA levels of TREK channels were measured on DRG cells treated for 6 h with 0.1 and 1.0 µM amiloride. We observed that the treatment with both the concentrations of amiloride significantly decreased neuronal pH_i_ and increased the transcript levels of TREK channels (Figure 2).

### 2.3. OHP-Induced Cytosolic Acidification Positively Regulates TREK-2 Channel Activity

The potassium background current mediated by the activation of K2P channels is a key determinant of the membrane resting potential (V_rest_). Several members of this channel family are expressed in DRG neurons, and TREK-2 and TRESK channels are the main contributors to V_rest_ in small- and medium-sized neurons in rats [22,23]. Moreover, intracellular proton concentrations regulate TREK channel activity in different ways: TREK-1 and TREK-2 are both activated by acidification, while TRAAK activity is stimulated by alkalization from pH 7.3, but without being affected by cytoplasmic acidosis [24]. Therefore, TREK-1 and TREK-2 are probably the most active background channel types in small- and medium-sized DRG neurons after OHP treatment.

We studied the behavior of TREK-2 channels in small-sized treated neurons (OHP 48 h) by performing single-channel patch–clamp experiments in cell-attached configuration to preserve the intracellular milieu composition. To achieve a stable membrane potential near 0 mV without inducing intracellular free calcium increases, we perfused DRG neurons with a bath solution containing 150 mM KCl and low [Ca^2+^]. Furthermore, the pipette solution contained TEA chloride, CsCl, and 4,4’-Diisothiocyano-2,2’-stilbenedisulfonic acid (DIDS) to minimize the contribution of channels other than K2P. Moreover, we identified TREK-2 channels by means of their distinctive biophysical properties. First, in mammalian neurons, TREK-2 exhibits two main channel phenotypes with small and large conductance, TREK-2S and TREK-2L, which can coexist in the same patch [25]. Both TREK-2S and TREK-2L channels are activated by low pH_i_ and arachidonic acid [25]. Finally, a single-channel TREK-2 current–voltage relationship showed a characteristic inward rectification in symmetrical potassium concentrations [25,26,27].

**Figure 3:**
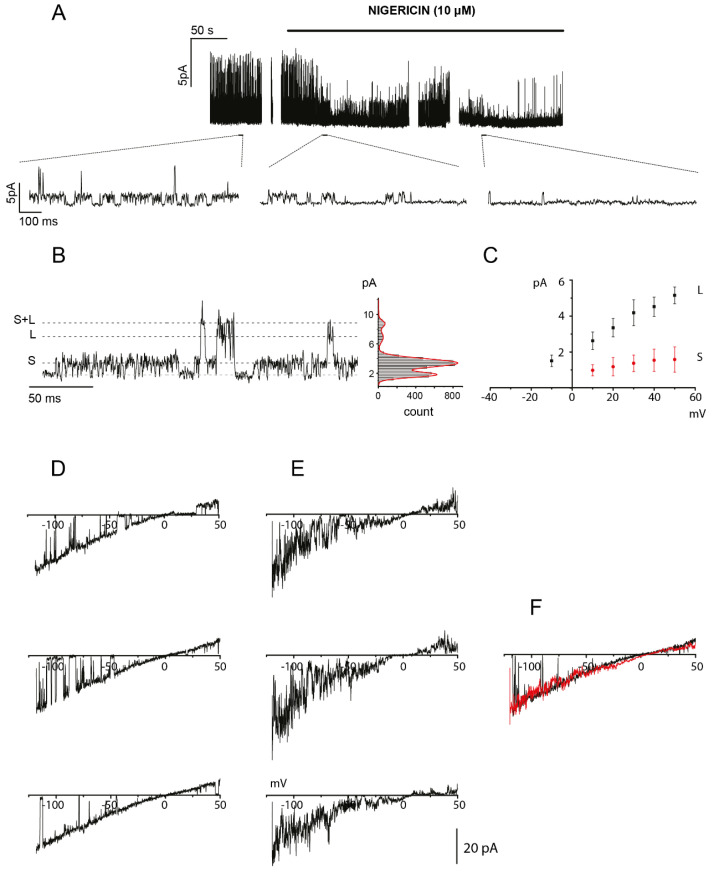
Oxaliplatin-dependent cytosolic acidification modulates TREK-2 channel activity. (**A**) Single-channel recording at V_hold_ = +40 mV from an OHP treated DRG neuron, before and during nigericin perfusion. When nigericin sets the pH at physiological levels, TREK-2 channel activity strongly decreased. Insets show a magnification in three different time recording points (2 min before nigericin perfusion; 1 and 4 min after nigericin perfusion, respectively). (**B**) An exemplary single-channel current trace recorded in cell-attached configuration at V_hold_ = +40 mV from an OHP treated DRG neuron and the corresponding amplitude histogram. Two different levels of current could be observed: the smaller one (S) and the larger one (L). (**C**) I-V plot for the channel levels S and L shown in (**B**). (**D**) Single-channel currents recorded during voltage ramps in an OHP-treated neuron in a cell-attached patch with high potassium solution in the pipette. (**E**) Exemplary single-channel current trace recorded in an inside-out configuration during the stimulation with arachidonic acid (2 µM) in symmetrical K^+^ solutions. (**F**) Merge of the average of 10 current ramps recorded in the presence of arachidonic acid (red trace) with a single-channel ramp curve (black trace) taken from the same sample of the traces shown in (**D**).

Figure 3A shows the single-channel currents recorded at a holding potential of 40 mV in an OHP-treated neuron before and during the perfusion of the high potassium solution containing 10 μM nigericin at pH 7.4. In these ionic conditions, nigericin was generated in neurons with an acidic pH_i_, a net efflux of H^+^ that set its value near 7.4 [13]. Following the perfusion with nigericin, the patch activity *NP_o_* decreased from 0.57 ± 0.19 to 0.08 ± 0.11 (*N* being the number of channels in the patch and *P_o_* the channel open-state probability), in agreement with a high sensitivity of these channels to acidic intracellular pH. The effect of nigericin perfusion was observed in *n* = 6 patches. Moreover, in the insets of Figure 3A, as well as in the trace with an expanded scale shown in Figure 3B, channel openings with a small (S) and a large (L) current amplitude can be observed indicated by dotted lines. Both channel phenotypes were observed in four patches, and the remaining two showed only the S or L phenotype. The best fit of the amplitude histogram gives a mean current level of 1.68 pA and 5.21 pA. Figure 3C, from the same experiment, shows the single-channel I-V relationship obtained by plotting the mean amplitudes of the two current levels at different membrane potentials. Because in these ionic conditions, the actual reversal potential (V_rev_) is very difficult to evaluate, we performed a series of experiments replacing 130 mM CsCl with 150 mM KCl in the pipette solution. These conditions allowed us to observe the inward rectifying behavior that characterizes TREK-2 channels [25,28]. The traces in Figure 3D represent examples of single-channel openings recorded in an OHP-treated neuron by applying a 500 ms voltage ramp from −120 mV to 50 mV, after baseline subtraction and plotted against the voltage. The single-channel I-V was slightly inwardly rectifying with a V_rev_ close to 0 mV and a single channel conductance at −50 mV of about 200 pS and +50 mV of about 140 pS. Finally, in inside-out experiments, such as the one shown in Figure 3E, bath addition of arachidonic acid (AA; 10 µM), a potent activator of TREK-2 [22], opened a channel with similar properties. This is well highlighted in Figure 3F, where the average current obtained from a series of 10 current ramps recorded during the administration of AA is superimposed (red trace) with a single ramp from the cell-attached experiment (black trace).

In conclusion, these data provide evidence that TREK-2 channels are present on the membrane and functional at 48 h post-OHP-treatment and that their activity is positively modulated by OHP-induced pH_i_ changes.

### 2.4. Effects of OHP on Electrical Activity in DRG Neurons

As already stated, the magnitude of the K^+^ leak current is a primary determinant of neuronal V_rest_ [5]. An increase in TREK channel expression and/or activity is expected to induce membrane hyperpolarization and decreased excitability. To gain insight into the relationship between OHP treatment and DRG electrical activity, we performed extracellular recordings of action potentials from DRG neurons cultured on multielectrode arrays (MEAs).

**Figure 4:**
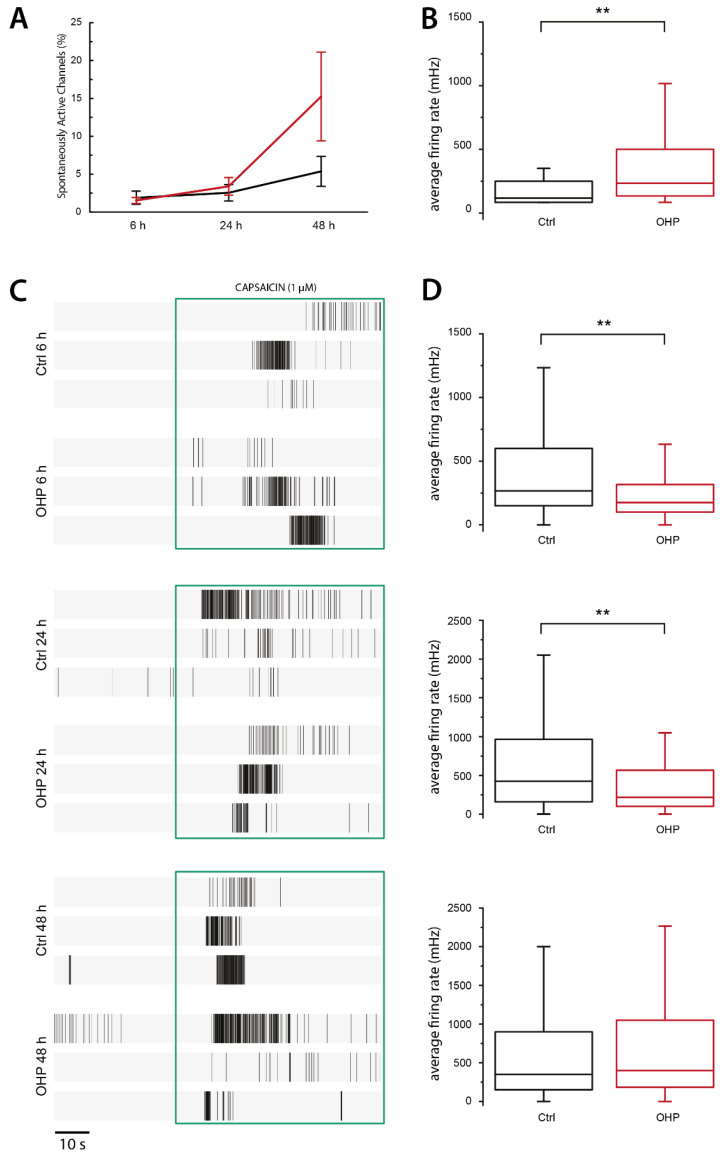
Effects of OHP treatment on spontaneous and evoked electrical activity in DRG neurons. (**A**) Trend of the percentage of basally active channels in control (black) and treated (red) conditions (mean ± SEM of *n* MEAs, with *n* ranging from 4 to 10). Data were analyzed through a linear regression of the log-transformed values, then the null hypothesis of zero-slope was tested. The percentage of spontaneously firing DRG neurons showed a positive trend in time when treated with OHP (*p*-value = 0.009) but not in control condition. (**B**) After 42 h from the treatment with OHP, the median firing rate per active channel resulted significantly higher (M_0,OHP_ = 233 mHz, IQR_0,OHP_ = [133; 500] mHz) compared to untreated condition (M_0,ctrl_ = 117 mHz, IQR_0,ctrl_ = [83; 250] mHz; ** *p*-value = 0.002, Mann–Whitney *U* test). (**C**) Three representative raster plots for each tested condition showing both spontaneous activity and extracellular action potentials (EAPs) detected during the application of 1 µM capsaicin (green box). (**D**) Box plots compare control (black) and OHP-treated (red) average spike frequency (in mHz) in the presence of capsaicin. After 6 h from cell plating, the capsaicin-induced median firing rate of the responsive channels was M_6,ctrl_ = 267 mHz, IQR_6,ctrl_ = [146; 600] mHz (86 channels, from *n* = 9 MEAs) and M_6,OHP_ = 175 mHz, IQR_6,OHP_ = [100; 321] mHz (90 channels from *n* = 9 MEAs) in control and treated conditions, respectively. After 24 h from cell plating, values were M_24,ctrl_ = 425 mHz, IQR_24,ctrl_ = [154; 967] mHz (72 channels, from *n* = 4 MEAs) and M_24,OHP_ = 217 mHz, IQR_24,OHP_ = [100; 567] mHz (96 channels, from *n* = 7 MEAs), in control condition and upon 6 h of OHP treatment, respectively. Finally, after 48 h from cell plating, values were M_48,ctrl_ = 350 mHz, IQR_48,ctrl_ = [150; 908] mHz (157 channels, from *n* = 6 MEAs) and M_48,OHP_ = 400 mHz, IQR_48,OHP_ = [183; 1050] mHz (155 channels, from *n* = 7 MEAs), in control condition and upon 6 h of OHP treatment, respectively. For all three comparisons, the Mann–Whitney *U* test was used and, at 6 h and 24 h, ** *p*-value < 0.01 (for exact *p*-values and effect sizes, see Table 1). In all the box plots, the box corresponds to the IQR, while the whiskers represent the minimum and maximum data points still within 2.2 IQRs off the lower and upper quartiles, respectively.

Previous studies have shown that DRG neurons, although representing a heterogeneous population of different sensory cells, have a quite limited and sporadic spontaneous electrical activity [29], which can, in any case, be detected by MEAs thanks to the high screening capacity. In this manner, the average number of spontaneously active channels and related firing rate could be measured in different conditions. In particular, three different time points were investigated: immediately after the removal of the treatment (OHP 6 h, *n* = 10 MEAs), 18 h later (OHP 24 h, *n* = 7 MEAs), and 42 h later (OHP 48 h, *n* = 7 MEAs). Untreated (control) cultures were tested in parallel after 6 h (*n* = 9 MEAs), 24 h (*n* = 4 MEAs), and 48 h (*n* = 6 MEAs) from cell plating. Each channel showing at least 5 spontaneous spikes within a time window of 60 s was considered basally active. Overall, the percentage of channels exhibiting some basal electrical activity increased significantly in time only in OHP-treated cells (*p*-value = 0.009, regression analysis), while the trend of the control cultures, albeit positive, did not show a significant slope (Figure 4A). It should be noted that, even if, in general, each single MEA electrode can detect extracellular action potentials (EAPs) coming from more than one cell (within a radius of tens of micrometers), the number of active channels is roughly proportional to the number of firing neurons in the culture. At 48 h, the latest time point explored, 15.3 ± 5.9% of the neurons treated with OHP showed spontaneous firing, while only 5.4 ± 2.0% of the channel was active in control MEAs. Notably, in addition to the increased number of spontaneously firing neurons, the median firing rate per active channel was significantly higher after 42 h from OHP treatment (M_0,OHP_ = 233 mHz, IQR_0,OHP_ = [133; 500] mHz) compared to the control condition (M_0,ctrl_ = 117 mHz, IQR_0,ctrl_ = [83; 250] mHz; *p*-value = 0.002, Mann–Whitney *U* test: Figure 4B).

To investigate the effects of OHP on neuronal susceptibility to external stimuli, we evoked transitory activity in DRG primary cultures by adding 1 µM capsaicin. EAPs crossing the voltage threshold were counted within a time window of 60 s in both OHP-treated and control conditions and for each of the three time points (Figure 4C). Table 1 shows the capsaicin-induced median firing rates and related interquartile ranges (IQRs) for each tested condition. In summary, OHP treatment elicited a statistically significant decrease in median firing rate compared to the control electrical activity, at both 6 h and 24 h (Mann–Whitney *U* test, *p*-value = 0.0018 and *p*-value = 0.0091, respectively), while no significant differences were observed at 48 h.

Taken together, these results suggest that OHP administration can exert an early and transient impairment of neuronal excitability, followed by a late recovery phase in which spontaneous firing is enhanced.

## 3. Discussion

Modulation of the expression of neuronal ionic channels by antineoplastic drugs has been suggested as a common mechanism of chemotherapy-induced peripheral neuropathy [30,31]. In this regard, the involvement of the thermo- and mechanosensitive TREK channels in OIPN is well documented [2,18]. These studies have highlighted a downregulation of TREK channel expression in nociceptors 90 h after OHP injection in mice and have suggested that such alteration contributes to cold hyperalgesia, cool allodynia, and mechanical hypersensitivity. Indeed, from a mechanistic point of view, lowering the expression of K^+^ background channels produces depolarization of V_rest_ and modifies neuron excitability. However, no data are present on the early effects of OHP treatment on the TREK channel expression. Data presented in this paper fill this gap by showing that therapeutically-relevant concentrations of OHP [13] induce an early transient overexpression of TREK-1, TREK-2, and TRAAK both in cultured DRG neurons and in DRG cells from treated animals. Furthermore, this alteration is relatively specific, as no changes in TRPV1 channel expression were observed. Since OHP induces early cytosolic acidification in sensory neurons, we tested whether lowering the intracellular pH produced a similar alteration of gene expression. Notably, following the inhibition of the activity of the NHE transporters with amiloride, which lowered pH_i_ to values similar to OHP treatment, we observed an upregulation of the expression of the TREK genes. This result suggests a possible direct role of low pH_i_ in TREK gene regulation. Interestingly, it has been reported in a rat model that TREK channel expression is enhanced in the cortex and hippocampus during acute cerebral ischemia [32], where, among other things, intracellular neuronal acidosis occurs [33]. How intracellular acidosis regulates gene expression has been determined in yeast and bacteria [34,35,36], but information concerning this effect on mammalian cells is scarce and fragmentary [37]. However, other scenarios could explain the amiloride effect on gene expression. Zhou et al. pointed to the role of NHE1, the most ubiquitous member of the mammalian Na^+^/H^+^ exchanger family [21,38], as a signaling protein involved in gene expression regulation [39]. In the brain of a spontaneous NHE1 null mutant mouse strain, several genes were significantly up- or downregulated. In particular, for one of them, the monocarboxylate transporter 13 gene, pharmacological inhibition of NHE1 produced a downregulation in primary cultures from wild type mice that was unrelated to pH_i_ acidification. Finally, it is worth noting that cisplatin, another platinum-based antineoplastic drug, acts as a noncompetitive inhibitor of NHE1 [40,41]. It would, therefore, be important to verify whether OHP, at the therapeutically-relevant concentration used in our study, shares this action, since this would provide a further plausible mechanism for the early acidification observed both in vivo and in vitro DRG treated neurons alongside hemoglobin binding that our group reported earlier [14].

In response to a pH_i_ decrease, TREK-1 and TREK-2 channels should increase their activity. The most effective way to characterize TREK channel responses to OHP-induced acidification is by recording single-channel current from cell-attached patches, a configuration that represents the method of choice to describe the ion channels without disturbance of the intracellular milieu in OHP treated neurons. We focused on the TREK-2 channel that is easily identifiable thanks to its unique biophysical properties and confirmed that in small-sized OHP-treated neurons, the channel open probability was strongly increased, as expected, and that this hyperactivity was inhibited by restoring pH_i_ to physiological levels.

In DRG neurons treated in vitro with oxaliplatin, we observed an early and transient negative regulation of both spontaneous and capsaicin-evoked electrical activity. In mice, TREK channels are highly expressed in both peptidergic and nonpeptidergic small sensory neurons [8,42]. Furthermore, TREK-1 extensively co-localizes with TRPV1 [8], the capsaicin-activated nonselective ion channel. Moreover, in the majority of neurons innervating urinary bladder and distal colon that express TRPV1, mRNA were also detected the gene transcripts of TREK channels, with the prevalence of TREK-1 mRNA [43]. In light of this, capsaicin is a good stimulus to study evoked firing in nociceptors, also because the expression of TRPV1 channels was unchanged by OHP-treatment. The negative regulation of neuronal excitability correlated well with the observed modulation in channel expression and pH_i_-dependent activity. First, the control of pH homeostasis is essential for the regulation of neuronal excitability [20,21], and intracellular acidification decrease DRG firing [44]. Moreover, the global effect of these changes likely consists of a transient increase in background currents that causes a membrane potential hyperpolarization making the cells less excitable; that is, larger depolarizations are required to elicit action potentials. In this regard, the lack of effect at 48 h may be related to the restoring of channel expression similar to control conditions. However, as many other channels types are targets of OHP modulation [30], we cannot exclude that additional contribution of other currents unbalanced the membrane properties toward increased excitability. Nevertheless, a clear understanding of the mechanism that regulates the expression of TREK channels in nociceptors can be useful to reveal novel therapeutic targets with the aim of preventing or treating OIPN.

## 4. Materials and Methods

### 4.1. Animals

BALB/c male mice aged 5–10 weeks upon arrival were employed (Envigo, San Pietro al Natisone, Italy) both for DRG culture preparation and in vivo studies. Animals were maintained as previously reported [45]. Care and husbandry of animals were in conformity with the institutional guidelines in compliance with national and international laws and policies. The study plan was approved by the Animal Ethics Committee of the University of Milano Bicocca and the University of Piemonte Orientale. The procedures were approved by the local animal-health and ethical committees (the University of Piemonte Orientale and University of Milan Bicocca; n. 004874/14) and were authorized by the national authority (Ministero della Salute; authorization number n. DB064.N.TGU). All mice were euthanized under deep isoflurane-induced anesthesia for cell cultures and with CO_2_ for in vivo experiments.

### 4.2. Chemicals

For in vivo studies, OHP solution was prepared as reported by Renn and collaborators [46] and used as previously described [45]. For in vitro studies, OHP (5 mg/mL stock solution, Sigma–Aldrich Inc., Milano, Italy), capsaicin (1 mM stock solution, Sigma–Aldrich Inc., Italy), amiloride (5 mM, stock solution, Sigma–Aldrich Inc., Italy) DIDS (4,4’-Diisothiocyano-2,2’-stilbenedisulfonic acid; Sigma–Aldrich Inc., Italy), BCTC (10 mM, stock solution, Sigma–Aldrich Inc., Italy), icilin (10 mM stock solution, Sigma–Aldrich Inc., Italy), AA (arachidonic acid; Sigma–Aldrich Inc., Italy), and nigericin (20 mM stock solution, Life Technologies, Monza, Italy) were used. These compounds, with the exception of capsaicin and AA (reconstituted in 100% EtOH and water, respectively), were dissolved in 100% dimethyl sulfoxide (DMSO) and stored at −20 °C, according to the manufacturers’ specifications. For each experiment, working concentrations of these drugs were freshly prepared by diluting them in their relative vehicle.

### 4.3. Isolation, Culture, and Treatment of Mouse DRG Neurons

DRG excised from adult BALB/c mice (5–10-week-old) were collected in a dish containing cold F12 (Nutrient Mixture F12 Ham) medium (Sigma–Aldrich Inc., Italy). After accurate de-sheathing, DRG were transferred into a sterile 35 mm dish containing collagenase from Clostridium hystoliticum 0.125% (Sigma–Aldrich Inc., Italy) and DNase (Sigma–Aldrich Inc., Italy) in F12 (Nutrient Mixture F12 Ham) medium and incubated at 37 °C. After 1 h incubation, DRG were triturated using a 1000 µL tip. Myelin and nerve debris were eliminated by centrifugation through bovine serum albumin (BSA) cushion. Cell pellets were re-suspended in Bottenstein and Sato medium (BS): 30% F12 (Nutrient Mixture F12 Ham medium), 40% DMEM (Dulbecco’s Modified Eagle’s medium, Sigma–Aldrich Inc., Italy), 30% Neurobasal A medium (Life Technologies, Italy), 100X N2 supplement (Life Technologies, Italy), penicillin 10 U/mL and streptomycin 100 mg/mL (Sigma–Aldrich Inc., Italy), supplemented with Recombinant Human β-NGF, Recombinant Murine GDNF and Recombinant Human NT3 (Peprotech, Rocky Hill, NJ, USA) and plated onto 24 mm glass coverslips pre-coated with laminin (Sigma–Aldrich Inc., Italy). The administration of OHP and amiloride was done 48 h after the isolation of DRG neurons, and all the experiments were performed from 54 to 96 h of culture.

### 4.4. Real-Time Quantitative PCR (RT-qPCR)

Total RNA was isolated from DRG cultures using TRI-Reagent^®^ and reverse-transcribed according to the manufacturer’s instructions (Im-Prom-II™ Reverse Transcription System, Promega, WI, USA). For the in vivo study, samples were taken from the experiment described in [13]. Until used, cDNA was stored at −20 °C. RT-qPCRs were performed on 96-well plates (CFX96™ Real-Time PCR Detection Systems, Bio-Rad Inc., Milano, Italy), in triplicate and fluorescence intensity assessed using the CFX96™ Real-Time PCR Detection Systems (Bio-Rad Inc.). The initial denaturation step was set at 95 °C for 10 min, followed by 40 cycles of amplification using this set of primers: TREK-1 5′-TCACTCTGACGACCATTGGA-3′ forward, 5′-GAGGATCCAGAACCACACCA-3′ reverse; mouse TREK-2 5′-CATCTGTGTGAGTCCCCAGG-3′ forward, 5′-GACTGCTGCTGTTGGAAGAG-3′ reverse, and mouse TRAAK 5′-GTGTGAGCCAGAAGAGCCT-3′ forward, 5′-GGTTGCTGCTATTGGTCCAG-3′ reverse; TRPV1 5′-CCTGCATTGACACCTGTGAG-3′ forward, 5′-AGAAGATGCGCTTGACAAATC-3′ reverse; 60 °C annealing temperature. Transcripts were normalized to the expression of ribosomal protein S18 mRNAs, and, for each gene, the relative threshold cycle (Δ*C_t_*) was calculated. The Δ*C_t_* of treated cells was compared to the Δ*C_t_* generated by control cells, and log_2_ fold change was calculated as the difference between them (log_2_FC = −ΔΔ*C_t_*).

### 4.5. Measurement of Intracellular pH in DRG Cultures by Epifluorescence Microscopy with BCECF

DRG neurons were plated onto 24 mm round coverslips and were incubated with 1 μM BCECF (Life Technologies, Italy) in Krebs–Ringer Buffer (KRB, 135 mM NaCl, 5 mM KCl, 0.4 mM KH_2_PO_4_, 1 mM MgSO_4_, 5.5 mM glucose, 20 mM 4-(2-hydroxyethyl)-1-piperazine ethane sulfonic acid (HEPES), pH 7.4) containing 2 mM CaCl_2_. After 15 min (room temperature), the cells were washed and re-suspended in KRB (pH 7.4). A Leica DMI6000 epifluorescent microscope equipped with an S Fluor ×40/1.3 objective was used. Cells were alternatively excited at 490/450 nm (monochromator Polychrome IV, Till Photonics, Kaufbeuren, Germany), and the fluorescent signals were collected every 10 s (Hamamatsu, Shizuoka, Japan); the experiments were controlled and images analyzed using MetaFluor (Molecular Devices, Sunny-vale, CA, USA) software. To obtain the intracellular pH value, the 525/610 nm emission fluorescence ratios were compared with the calibration curves arising from the in vivo pH equilibration using the proton ionophore nigericin (10 µM) and Intracellular pH Calibration Buffer Kit (pH 7.5–5.5, Life Technologies, Italy).

### 4.6. Electrophysiology-Multielectrode Arrays (MEAs)

DRG extracellular action potentials (EAPs) were recorded by means of a commercial 60-channel multielectrode array (MEA) setup by Multi Channel Systems MCS GmbH. Specifically, a TC02 external temperature controller was used to keep the socket surface at 37 °C, while voltage signals were digitally acquired through a USB-ME64 digitizer unit at the sampling rate of 25 kHz. To eliminate slow fluctuations of the baseline and possible artifacts, such as those sporadically introduced by the perfusion system, traces were filtered using a 4-pole Bessel high-pass digital filter (cutoff frequency f_c_ = 100 Hz) as implemented in MC Rack software (Version 4.6.2, Multi Channel Systems MCS GmbH, Reutlingen, Germany). Then, the same software was used for EAP spike detection by setting a channel-specific voltage threshold equal to 5 times the standard deviation of the baseline noise (15 µV being the typical peak-to-peak noise level). Both upward and downward spikes were analyzed, but for every single channel, just one polarity—i.e., the most frequently occurring one—was considered for the subsequent analysis, to avoid multiple counting of possible biphasic spikes. NeuroExplorer software (Nex Technologies, Colorado Springs, CO, USA) was finally used to draw the raster plots of the spikes detected in each channel. For the entire duration of the experiments, DRG neurons were continuously super fused (≈2 mL/min flux) with a standard physiological solution (see composition in the next section about the patch–clamp technique), with the possibility to switch to a 1 µM capsaicin-containing solution to evoke neuronal firing activity. Capsaicin was perfused for 60 s, and the number of EAPs detected in that temporal window was used to compute the average firing rate induced by the compound. In each experiment, the 60 s immediately preceding capsaicin administration were used for spontaneous activity evaluation. In general, a channel was considered active—and suitable for firing rate analysis—only when it recorded at least 5 spikes within one of the two aforementioned 1-min windows.

### 4.7. Electrophysiology—Patch–Clamp

Cell attached and inside-out patch–clamp recordings were performed at 22–25 °C on control and OHP-treated neurons with a mean soma diameter <25 μm. During gigaseal formation, DRG neurons were continuously super fused with a standard physiological solution of the following composition (in mM): NaCl 154; KCl 4; CaCl_2_ 2; MgCl_2_ 1; 4-(2-hydroxyethyl)-1-piperazine ethane sulfonic acid (HEPES) 5; glucose 5.5; NaOH to pH 7.4. The resistance of patch electrodes, prepared from borosilicate glass capillaries (World Precision Instruments), was 5–7 MΩ. In most cell-attached experiments, pipette solution composition was (in mM): CsCl 130, TEACl 20, DIDS 1; 4-(2-hydroxyethyl)-1-piperazine ethane sulfonic acid (HEPES) 10, EGTA 2, BCTC 3 μM, Icilin 1 μM, CsOH to pH 7.4. In the second set of experiments, the pipette solution composition was (in mM): KCl 130, TEACl 20, DIDS 1; 4-(2-hydroxyethyl)-1-piperazine ethane sulfonic acid (HEPES) 10, EGTA 2, CsCl 1, KOH to pH 7.4. Once the seal was obtained (2–10 GΩ), cells were perfused with a solution containing KCl 150, MgCl_2_ 2, CaCl_2_ 1, EGTA 1.1, HEPES 5, KOH to pH 7.4, to set the membrane potential near 0 mV and to prevent intracellular calcium loading. Inside-out experiments were performed on untreated DRG neurons. The pipette and the bath solutions were the 130 mM KCl solution described above. Data were collected and filtered at 1 kHz with an Axopatch 200B amplifier (Molecular Devices, San Jose, CA, USA) and continuously digitized at 1 kHz sampling frequency with PClamp Axoscope software (Molecular Devices, USA). Steady-state voltage-clamp protocols were applied and digitized at 10 or 20 kHz with PClamp Clampex software. Data analysis was performed with OriginPro (OriginLab, Northampton, MA, USA) and PClamp Clampfit software.

### 4.8. Statistical Analysis

Normally distributed data were expressed as mean ± standard error of the mean (SEM), and parametric hypothesis tests were used whenever possible, after having checked for dataset homoscedasticity and residual normality (Levene’s and Shapiro–Wilk tests, respectively). In particular, unless otherwise specified, two-sample comparisons were performed using the unpaired two-tailed Student’s *t*-test, while, for comparisons between more than two experimental groups, analysis of variance (ANOVA) was used along with HSD Tukey or Dunnett’s post hoc test. When the above assumptions were not met, nonparametric alternative tests were used as detailed in the text, and median (M) and interquartile range (IQR) were used as measures of central tendency and dispersion, respectively. In all cases, tests were conducted at the significance level α = 0.05. Single-channel current amplitudes were represented as mean ± standard deviation (SD), estimated by fitting the amplitude histograms to a sum of Gaussian functions (OriginPro 9.1, OriginLab, USA).

## Figures and Tables

**Table 1:** Descriptive and inferential statistics about the electrical response of dorsal root ganglion (DRG) neurons to 1 µM capsaicin administration. M is the median of the average firing rate computed over a 1 min-window, starting from the beginning of the agonist perfusion. IQR, Q1, and Q2 are the interquartile range, the first and the third quartiles, respectively. Below are the number of capsaicin-responsive channels and the number of independent multielectrode arrays (MEAs) from which these channels come. For each time point, *p*-values refer to the independent sample Mann–Whitney *U* test. Finally, for each time point effect, size is given in terms of both correlation coefficient *r* and Cohens’s *d*.

	6 h		24 h		48 h	
	Ctrl	OHP	Ctrl	OHP	Ctrl	OHP
M (mHz)	267	175	425	217	350	400
IQR (mHz)	454	221	813	467	758	867
Q1 (mHz)	146	100	154	100	150	183
Q3 (mHz)	600	321	967	567	908	1050
*n* channels	86	90	72	96	157	155
*n* MEAs	9	9	4	7	6	7
*p*-value	0.0018		0.0091		0.2350	
*r*	0.2348		0.2012		0.0672	
Cohen’s *d*	0.1657		0.3872		0.0670	
